# 

**Published:** 1889-03

**Authors:** 


### CONTENTS.

### COMMUNICATIONS.

When is Extraction a Necessity? ...... 109
Characteristics of Alloys ...... 112
The Chicago Dental Society ...... 117
MONTHLY DIGEST FOR FEBRUARY ...... 140

### SELECTIONS.

Disease in Adults and in Children ...... 147
The Air of the Bed-Room ...... 148
Skin-Grafting ...... 150

### EDITORIAL.

The Chicago Dental Society ...... 152
Dental Protective Association ...... 153
Educational ...... 154
St. Louis Dental Society—Programme ...... 157
Biographical ...... 158
Waste no Time ...... 160

